# Quantitative Measurements of Autobiographical Memory Content

**DOI:** 10.1371/journal.pone.0044809

**Published:** 2012-09-21

**Authors:** Robert S. Gardner, Adam T. Vogel, Matteo Mainetti, Giorgio A. Ascoli

**Affiliations:** 1 Center for Neural Informatics, Structures, and Plasticity, Krasnow Institute for Advanced Study, George Mason University, Fairfax, Virginia, United States of America; 2 Psychology Department, George Mason University, Fairfax, Virginia, United States of America; 3 Molecular Neuroscience Department, George Mason University, Fairfax, Virginia, United States of America; University Of São Paulo, Brazil

## Abstract

Autobiographical memory (AM), subjective recollection of past experiences, is fundamental in everyday life. Nevertheless, characterization of the spontaneous occurrence of AM, as well as of the number and types of recollected details, remains limited. The CRAM (Cue-Recalled Autobiographical Memory) test (http://cramtest.info) adapts and combines the cue-word method with an assessment that collects counts of details recalled from different life periods. The SPAM (Spontaneous Probability of Autobiographical Memories) protocol samples introspection during everyday activity, recording memory duration and frequency. These measures provide detailed, naturalistic accounts of AM content and frequency, quantifying essential dimensions of recollection. AM content (∼20 details/recollection) decreased with the age of the episode, but less drastically than the probability of reporting remote compared to recent memories. AM retrieval was frequent (∼20/hour), each memory lasting ∼30 seconds. Testable hypotheses of the specific content retrieved in a fixed time from given life periods are presented.

## Introduction

Autobiographical memories (AMs) are recollections of the first-person experience of past episodes. They refer to spatially and temporally specific events rather than factual (semantic) knowledge about the world [1]–[3]. From the neuropsychological perspective, AMs are long-term, i.e. the potential for their retrieval lasts from minutes to the entire life span, and are distinguished in the underlying brain organization from short-term or working memories [4]–[7].

AMs are believed to subserve fundamental thoughts and behaviors [8], [9] and important dimensions of autobiographical recall have been extensively characterized. Some psychophysical quantities have been measured objectively, such as response time [10], [11]. Among other aspects, emotional level, importance, and rehearsal have been rated on a Likert scale [12], [13]. In addition, AM accuracy, intensity, and retrieval efficacy have been probed by systematically documenting daily events over extended periods of time [14]–[16].

The temporal distribution of AMs has also been studied comprehensively. Galton [17] initially described a systematic protocol for eliciting his own AMs in order to sample their distribution over his life span. Crovitz & Schiffman [18] revised this method with the introduction of the cue-word technique: single words are sequentially presented to participants as prompts for generating memories, which are labeled for later recall and subsequently dated to when the recalled event had occurred. An effect of specific cue words on the resulting temporal distribution was soon documented [19], and the consequent adoption of that same fixed word set in following studies created a *de facto* standard protocol of this cue-word technique [10], [11], [20]–[22]. The resulting studies yielded a reliable characterization of three components of the temporal distribution of AMs: the retention function, the reminiscence bump, and childhood amnesia. Retention of AMs declines steeply according to a power decay backward from the present day [21], [23]. The reminiscence bump is an increase in the relative recall of episodes that occur between 10 and 30 years of age [24], [25], and is best observed in adults older than 45 years. Childhood amnesia is a drastic reduction of episodes recalled from 0 to 4 years of age [26]. Notably, these characteristics are considerably robust to aging [11], pathology [27], [28], the sensory modality used to elicit recollections [29], [30] and the cueing technique [28].

Despite the history of cognitive and clinical research, certain dimensions of AM, namely the number and types of details comprising the recollection (i.e. content) and its spontaneous rate of occurrence (i.e. frequency), have received relatively little attention. Nevertheless, these dimensions of autobiographical recollection play pivotal roles in AM theory. For example, Conway & Pleydell proposed a hierarchical organization of AM termed the *autobiographical knowledge base* ([31]; also see [32]). This theory places abstracted thematic knowledge and summarized events at the top, and specific details of distinct episodes rich with context at the bottom. The dynamic relation between these components would support reminiscence: abstracted knowledge comprising one's life story is constrained by the sensory-perceptual and contextual details of individual events; in turn, storage of episodic details in long-term memory and their retrieval are influenced by abstracted knowledge. Several hypotheses on the relationship between the amount and types of details from individual events and the organization of knowledge structures according to certain features (e.g. person, place, feeling, time) [33], emerge from this model. For example, the amount of detail for selected features could predict the probability that an AM will be incorporated into certain knowledge sets expounding how episodic detail and abstract knowledge come together during memory storage and retrieval.

AM content also features prominently in theories of *source monitoring*, i.e. the process of retrieving and assigning the information in a memory to the original source (e.g. person A or person B). Under a framework set forth by Johnson and Raye ([12]; also see [34], [35]), evaluating the amount and type of recalled detail (e.g. emotion, location, time) is key for accurate source determinations. When the detail of various events (e.g. real and imaged) is uncharacteristic, mistakes may arise. Misattribution of select details can have drastic consequences, e.g. as observed in eyewitness testimony, and pathology [35].

The importance of both AM content and frequency is also highlighted in cross-sectional research. Aging and major neurodegenerative diseases selectively impair episodic and contextual memory [36], as compared to e.g. semantic memory [3]. This observation also applies to recollection of AM content [27], [28], [37], [38], including source monitoring [39], [40]. Similarly, the number of AMs elicited by the cue-word method, extracted from participant narratives or collected through participant diaries [41] also declines in aging and pathology [27], [28]. Moreover, specificity of AM content, when preserved in aging, is suggested to be a result of frequent rehearsal [40], [42]. Altogether, theories on AM structure and function, and age-related and pathological impairment, identify content and frequency as significant AM dimensions, urging their comprehensive measurement through the life span.

Several methods have been utilized to characterize aspects of AM content. The autobiographical memory interview was developed to assess the extent of what can be remembered by amnesic patients [43]. The memory characteristics questionnaire (MCQ) [12] and the autobiographical memory questionnaire [44] used Likert scales to rate, among other features, spatial and temporal specificity, vividness, and sensory detail. Subjective content has also been quantified by counting the number of categorical details (a measure complementary and distinct to rating scales [45]) in written or spoken narratives of recalled autobiographical events [37], [39], [46]–[48] or experimentally created “autobiographical” episodes [38], [39].

To the best of our knowledge, the rate of spontaneous AM occurrence has not been measured. A widely used method that holds potential to reveal the frequency of typical AMs provides participants with journals to document and annotate AMs as they occur in daily life. Several such diary-based studies investigated differences between voluntary and involuntary AMs. Voluntary AMs are retrieved deliberately, and involuntary AMs, while consciously recollected, are retrieved without intention. Although research designs included both qualitative (e.g. content ratings) and quantitative (e.g. temporal distributions, counts of detail) measures, a focus on voluntary-involuntary AM comparison precluded frequency assessment. Specifically, these studies limited daily AM documentation [41], [49], [50], influenced voluntary AM retrieval [49]–[51] or did not collect a precise time window of diary utilization [52].

Here we report quantitative measurements of content and frequency of everyday AMs from numerous life periods, obtained using two new naturalistic methods: 1) The Cue-Recalled Autobiographical Memory (CRAM) test, and 2) The Spontaneous Probability of Autobiographical Memories (SPAM) protocol.

The CRAM test elicits AMs using a word-set designed to replicate everyday written and spoken language cues. Participants are then asked to identify the age of each cued AM, and count the number of identifiable details within specified categories, similar to those investigated in the AM literature and highlighted as important components in AM theory [12], [34]–[36], [37], [39], [53], e.g. temporal and spatial details, persons, objects, and emotions. Such categories have also been shown to be age-sensitive (e.g. [37], [38]). CRAM adapts the cue-word method [18] and combines it with content assessments, asking the participant to count the occurrence of different features in an AM. This approach offers several advantages over previously used methods. The novel cue-set, designed to mimic natural language cuing experiences, should elicit AMs more closely comparable to those retrieved under real-life conditions. Moreover, using participant-counts rather than time-intensive experimenter-scored narratives [37]–[39], [46] greatly reduces the data collection workload. This permits analyses of more numerous AMs, thus increasing representation and temporal resolution across the life span. Much like seminal studies of AM temporal distribution [18] reliably quantified AM retention [54], counts of feature-specific details of AMs naturalistically sampled across the life span can provide the foundation for quantitatively characterizing feature-specific retention. This methodology has already been adapted to an internet-based application (http://cramtest.info).

The SPAM protocol expands experience sampling techniques to measure the probability and duration of AM recall during everyday life, yielding estimates of the number of AMs experienced in a given time period. SPAM was inspired by and adapted from an original experiment by Brewer [55] in which participants carried a buzzer that prompted them at random times to annotate their behavioral and mental states and surrounding events for later analysis. This approach has been employed, among other applications, to assess visual activities [56] and psychopathology such as mood disorders [57]. The technique provides several advantages over assessments performed in clinical settings, including real-time monitoring, elimination of retroactive reporting errors, and performance evaluation in the natural environment [58]. In addition, this technique reduces the participant workload as compared to a strictly diary-based research design. To our knowledge, SPAM is the first application of experience sampling to measure AM probability and duration, resulting in the first quantification of spontaneous AM frequency.

Combined data from CRAM and SPAM enable previously inaccessible estimations related to AM recall, e.g. the average number of different features (people, feelings, etc.) recalled from distinct life periods in a given time window. Such a computation assumes that the temporal distributions and content of naturalistically-cued experimental AMs and everyday occurring AMs are similar. While this assumption is untested, the resultant predictions provide a quantitative base for experimentally testable hypotheses about the recall probability of defined subjective content from past life periods. Such a comprehensive characterization is necessary to inform theoretical and computational models of AM [54] and provides the groundwork to test how AM content and frequency change with age, pathology, and differing physiological conditions.

## Methods

### CRAM

CRAM is comprised of four parts delivered using computerized interactive forms: (Part 1) Non-identifiable information, i.e. month and year of birth, gender, and whether English is a native language, is collected from the participant; (Part 2) Thirty word-cued AMs are uniquely labeled by the subject with brief text descriptions; (Part 3) The text descriptions of each memory are re-presented one by one for the participant to date the recalled event; (Part 4) The text descriptions of a subset of the dated memories are re-presented once again, one at a time, for the subject to score each AM for content by counting the number of items in the recollection in each of eight specified categories.

The scope and details of how CRAM cues and scores AMs are substantially different from previous methods [18], [19], [28], [37]–[39], [46]. Thus, the full written instructions of these sections of CRAM (parts 2 and 4) are provided in the Supporting Information S1 (sections A and B–C, respectively). These scripts, which remained fixed throughout the study, were progressively developed with the aid of debriefing interviews during extensive preliminary experiments (not reported here).

#### Memory definition, cueing, and word sampling

In part 2, participants were provided with the following **DEFINITION**:

**Autobiographical memories are recollections of past episodes directly experienced by the subject. These memories should be of a brief, self-consistent episode of your life. An episode can be as short as a single snapshot and up to a few seconds long.**

**Figure 1:**
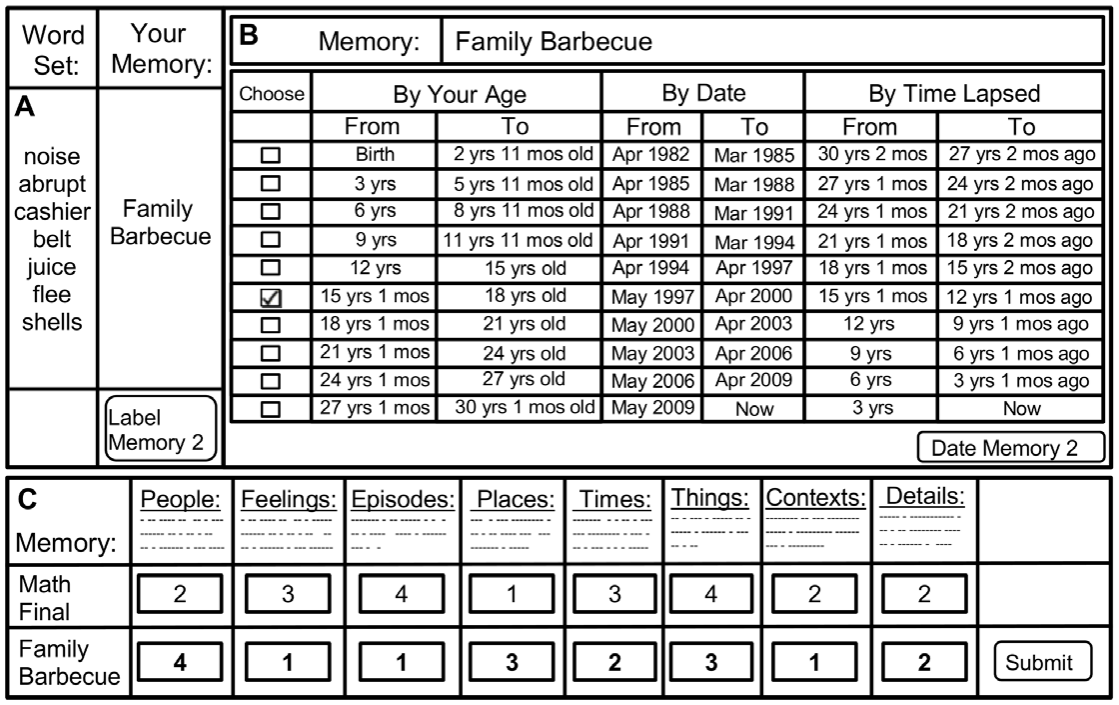
Graphical appearance of the Cue-Recalled Autobiographical Memory (CRAM) test user interface. Each panel represents a separate part of the CRAM test. (A) Subjects first label 30 memories recalled upon presentation of 7 words stochastically sampled from their natural language usage frequency. (B) Each memory is then dated into one of 10 temporal bins, based on the subject's age at the time of the event, the date of the event, and/or the time lapsed from the event. (C) Finally, participants score the content of 10 memories by counting the number of elements recalled from the event for each of eight distinct features (People … Details). Every feature is accompanied by a brief definition, schematically illustrated here by a few dotted lines underneath (see section B of the Supporting Information S1 for their full text).

Limiting the duration of the recalled episode to a few seconds allows for segmentation of multiple recalled events [59], which facilitates scoring of just one episode rather than a combination of many recollections. Subjects were instructed to read through a set of 7 word cues, to identify the first AM that came to mind, and to label it with either a unique word or phrase. Any single (or group of) cue-word(s) from the set could be used to trigger an AM. If no memory was elicited, participants were able to call up a new set of words, until 30 AMs were successively generated and labeled for later recall. Two major differences distinguish CRAM from the commonly adopted standard cue-word method [19]. First, each cue consisted of a list of 7 words rather than individual words, a number determined by trial and error in early pilot experiments that enabled relatively quick and probable autobiographical recall. Second, the words were not selected from a small, fixed sample as in many previous studies (e.g. [18]–[22]). Rather, they were chosen randomly from the 100,000,000-word British National Corpus [60] (see section D of the Supporting Information S1 for processing details), a compilation of written and spoken works. Therefore, word-set sampling was based on natural usage frequency, and differed dynamically from subject to subject (see e.g. Fig. 1A). The rationale for these choices was to achieve a sampling of AMs as close as possible to those occurring under normal circumstances, as elicited e.g. by everyday conversations, readings, or one's internal dialogue.

#### Memory dating

In part 3, the participant's age was used to divide their life span (e.g. [61], [62]) into 10 equal temporal periods or bins, numbered 0 to 9. We use the term *youth* in reference to the age of the subject at the time of the recalled episode relative to their age at the time of study participation. Three equivalent yet complementary methods were made available to participants to help them allocate each memory to one of the youth bins (Fig. 1B): subject's age (e.g. from 15 years and 1 month old to 18 years old), date of event (e.g. from May 1997 to April 2000), and time lapsed (e.g. from 15 years and 1 month ago to 12 years and 1 month ago). Participants were instructed to use (and encouraged to switch between) the method(s) that best helped them accurately date each memory. Given that accurate dating of select memories may be difficult, subjects were able to assign AMs to multiple bins, if needed. This option also addresses the possibility that temporal bins based on the subject's age might create cutoffs intersecting a temporal range associated with a particular episode. Multiple bins were selected for 2.8% of all dated AMs. During subsequent analyses, when applicable, AMs were weighted according to the number of bins to which they were assigned. The first 2 AMs were considered practice with the procedure and excluded from further processing.

#### Count of elements within specified memory features

In part 4, a subset of the 28 dated memories was pseudo-randomly selected for content scoring. Participants using an earlier version of the interface (implemented in Microsoft Excel) scored one AM from each reported bin. Participants using the later version (running in regular internet browsers) scored 10 AMs; one memory from each reported bin, plus if applicable (i.e. if not all bins were represented by a subject) additional AMs were selected starting in order from the least to most represented bins in the entire data base for all memories across subjects at that point in time. This latter procedure maximizes coverage of scored AMs across bins, while relaxing the constraint of uniform bin coverage for each subject. The labels of the sampled memories were re-presented one by one in random order. Participants were asked to count as many details as they could recall for each memory with the following instructions:

In this part you will revisit your recorded memory. For this memory, your task is to count how many elements you remember. There are 8 categories of elements, each with a short description and example - click on the category's name to see the example. Once you have counted the elements of a given category, enter that numerical value in the proper box, and proceed to the next category. After completing all categories for a memory, press ‘submit’ to display the next memory. Click *here* for additional guidance on what constitutes ‘an element’.

In this paper, we refer to details as *elements*, their categories as *features*, and the summed element counts for all features as *total content*.

The eight specified features were *Contexts*, *Episodes*, *Feelings*, *People*, *Places, Things*, *Times*, and (other) *Details*. These CRAM features are similar to those investigated in the AM literature. For example, a meta-analysis of 84 articles on episodic memory [36] compiled a categorical list of commonly characterized variables. These included descriptors of temporal sequences (*Episodes*), events (*Contexts*), temporal specificity (*Times*), perceptual features (*Details*), objects (*Things*), self (*Feelings*), persons (*People*), and spatial features (*Places*).

Although all features were displayed together, their order was randomized for each participant (and kept fixed for the ∼10 scored AMs). The description of each feature was in the form of a question that remained visible throughout the scoring section. However, the subject had the option to collapse or expand back the descriptions at any point after the first scored memory. An example of how elements should be counted for each feature was also offered through a clickable link. The descriptions and clickable examples are provided for each of the features in section B of the Supporting Information S1. Participants were offered the option of additional guidance on whether an element should be counted within a particular feature by means of a hyperlink (which remained available throughout the scoring section) to a detailed explanation, reported in section C of the Supporting Information S1. According to the test logs, individual feature-counting examples and general additional guidance were invoked on average 1.10 and 0.12 times per participant, respectively. All instructions and additional guidance were written to minimize biases or priming effects. In particular, the content of the examples focused on the definition of the respective feature, avoiding references to specific life periods (except for the *Times* feature), and traumatic or important episodes.

#### Graphical user interface, implementation, and availability of CRAM

All components of the CRAM test, including the word sampling algorithm, graphical user interface (GUI), and the response-driven transitions within and between the four parts, were developed and deployed in two separate formats and environments. One was based on Microsoft Excel and implemented in Visual Basic, while the other was based on standard internet browser protocols (HTML) and implemented in PHP/Java script (Fig. 1). Each of these two versions was complete, independent, and fully functional. Although the “touch and feel” of the two GUIs was different, the exact wording and sequential order of the functions were identical.

All results described in this paper are derived from a procedure in which subjects took the test on a local computer in the lab with one of the investigators present in the room. A version of the CRAM test for internet browser was later adapted, and is currently available for online use (http://cramtest.info) [63]. A version of the Excel implementation has also been developed in Italian, with faithful translation and based on an established 500,000 word spoken Italian corpus (http://badip.uni-graz.at). All versions of CRAM are available from the corresponding author upon request.

#### Participants, data screening, and analysis

The subject pool consisted of George Mason University undergraduate students recruited through the Psychology Department's enrollment web site. Students received course credit for successful study completion. This research was approved by the George Mason University Human Subject Review Board in accordance with Federal regulations and Mason policies for the protection of human subjects. Written consent for participation was obtained prior to data collection. All reported data are from subjects between 18 and 36 years of age (mean ± standard deviation: 21.17±3.77; median: 20). Memory dating involved 111 participants (83 females, 28 males; 72% native speakers) using the Excel format and an additional 83 participants (63 females, 20 males; 74% native speakers) using the web browser format. Out of the 5,432 dated memories, 1,424 memories were scored for content by 103 participants (77 females, 26 males; 75% native speakers) using the Excel format plus 79 participants (61 females, 18 males; 72% native speakers) using the web browser format.

All data were stored in a relational database (MySQL 5) and queried for quantitative measurements in SQL language. The extracted parameters were imported in R [64], SPSS, and Excel for statistical analysis and graphical output. Multiple tests of probability were corrected using the Bonferroni method. Data were initially inspected by the investigators to ensure the reasonable authenticity of the responses and to minimize the impact of intentional hoax, lazy entries or honest typos. Representative screening examples are reported in section E of the Supporting Information S1. This process resulted in the exclusion of data from 5 subjects plus 54 individual memories.

### SPAM

The SPAM protocol was devised to estimate the “Spontaneous Probability of Autobiographical Memories” by measuring the probability and duration of naturally occurring AMs during the course of everyday life using experiencing sampling [55]. The SPAM procedure randomly prompts participants at specific instants during the day to note whether in those very moments they are recalling an AM. When they are in fact experiencing an AM, they are asked to estimate the length of time of their reminiscence up to the point of the prompt. From these data, the fraction *f* of random prompts that correspond to an AM event is obtained by dividing the AM-associated prompts by the total number. Moreover, the duration *d* of an AM is computed by doubling the time estimate of the reminiscence, because on average the prompt interrupts the middle of the AM. The number *N_t_* of memories that are spontaneously recalled in a given period *t* can be estimated as *N_t_ = f • t/d*. In this formula, *f* represents the probability of experiencing an AM at any one moment in time, and *t/d* corresponds to the total number of possible AMs experienced in a given period of time. Their product (*f • t/d*) captures the number of memories in a temporal window, given a participant-specific probability of occurrence and average duration.

The protocol design capitalizes on the widespread technology of mobile telephony. An auto-dialer program was custom written for a computer modem to randomly call participants within variable constraints. Participants were given a choice of the number of daily calls they would receive and the hours to exclude from calls for sleep or other reasons. As the goal of SPAM is to measure the frequency of typically occurring AMs independent of retrieval mechanism, no distinction was made between voluntary and involuntary memory.

Upon initial briefing, SPAM participants were given a packet containing the informed consent form, a concise description of the protocol, a log booklet, and a form to record general biographical information and calling parameters (e.g., number of calls allowed per day). The packet also included the definition of AM as well as examples of mental states that should or should not count as AMs. The text of these examples is reported in full in section F of the Supporting Information S1. One of the investigators verbally reviewed the contents of the packet with all participants. When subjects received a call, they were instructed to perform a mental check on whether they had been experiencing an AM at that very moment and to write on the log book their best estimate of the memory duration up to the point of the phone call. Otherwise, a dashed line was used to indicate the absence of an AM event at the time of call. Subjects were encouraged to program a specific ring-tone for the number used by the auto-dialer to allow for a more instant reaction to the prompt; alternatively, SPAM calls were identified by caller ID.

#### Participants, data screening, and analysis

The subject pool consisted of George Mason University undergraduate students recruited through the Psychology Department's enrollment web site. The pool of subjects was the same as used for CRAM, however, the participant samples were entirely non-overlapping. Students received course credit for successful study completion. The protocol was approved by the George Mason University Human Subject Review Board in accordance with Federal regulations and Mason policies for the protection of human subjects. Written consent for participation was obtained prior to data collection. A total of 53 subjects underwent testing, and 16,801 phone calls were made altogether. On average, subjects received 17 calls per day (range 8–22) and selected daily calling windows of 11 (range 5–18) hours. The mean number of calls received by each participant over the course of the entire experiment was 317, with a standard deviation of 58 (range 184–480). The exact numbers depended on individual choices of parameter settings and an additional random factor due to the stochastic nature of the calling algorithm. For a typical subject, the SPAM experiment lasted an average of 19 (range 14–34) days. All reported data are from subjects (29 females, 24 males; 77% native speakers) between 18 and 37 years of age (mean ± standard deviation: 22.25±3.85; median: 21). Data were collected, entered in Excel for analyses, and excluded if values were more than 3 standard deviations from the mean, resulting in the exclusion of one data point pertaining to memory rate per hour (*N_hour_*).

## Results

### Memory Content is More Resilient to Temporal Decay than Retrieval Probability

**Figure 2:**
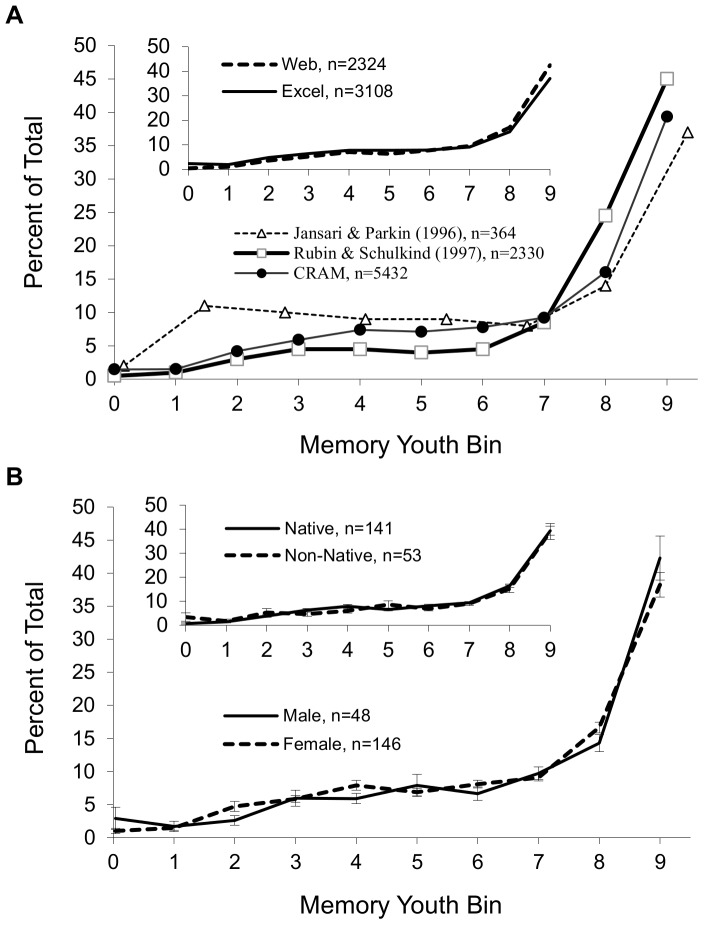
Temporal distributions of memories collected with the CRAM test. (A) Consistent with previous findings, the CRAM temporal distribution of autobiographical memories shows a retention effect in the most recent time bins and a power decay toward remote bins. Data re-plotted (with permission) from previous studies, i.e. Jansari & Parkin [20], and Rubin & Shulkind [11], are adapted by converting memory age to youth. The term youth reflects the age of the subject at the time of the recalled episode relative to their age at the time of study participation, and is grouped into 10 bins from the most remote (0) to the most recent (9) episodes. Temporal distributions are also equivalent between the Excel and web-based CRAM test formats (inset). (B) Temporal distributions, plotted as mean ± standard error across individual subjects to enable statistical comparison, are not significantly different between genders and between native and non-native English speakers (inset).

The temporal distribution of memories collected with CRAM is shown in Figure 2. More than 50% of AMs referred to episodes that occurred in the most recent 20% of the subject's life. This result quantitatively and qualitatively reproduces previous seminal findings using similar protocols (Fig. 2A). The reminiscence bump in the data of Jansari & Parkin [20], and its absence in ours and those of Rubin & Schulkind [11], are consistent with the participant age pools. Specifically, to discriminate the bump from the retention function, AMs must be sampled from time periods between these two phenomena. As the age of our sample falls within the constraints of the reminiscence bump, it is expected to be occluded by the retention function [20], [65]. Temporal distributions between the two CRAM interfaces (Excel- and web browser-based) were nearly identical (Fig. 2A inset). Also in agreement with earlier literature, the same distribution was observed for males and females (Fig. 2B), and for native and non-native English speakers (Fig. 2B inset). Overall, our analysis confirms the robust nature of this temporal distribution of AMs and its general independence of experimental details.

The average total content of AMs was the sum of elements from every feature computed as a weighed mean over all bins. The weight of each memory was its retrieval probability according to the temporal distribution of AMs, thus correcting for the sampling procedure that selected AMs for scoring equally between bins. For example, more recent AMs had greater weights based on the relatively large proportion of cued AMs occurring in more recent life periods. Given this formulation, a typical AM contained ∼20 elements. Although total content varied across individual memories (coefficient of variation ∼0.5), the average values were extremely similar between genders, graphical interfaces, and native/non-native English speakers (Table 1).

**Table 1:** Total content from all 1424 scored memories reported as the sum of elements from all features of each memory.

	Total	Test Format		Gender		English	
		Web	Excel	Female	Male	Native	Non-Native
n	1424	756	668	1095	329	1059	365
Mean	20.07	20.53	19.68	20.18	19.68	20.41	18.94
SD	9.82	10.17	9.40	9.84	9.76	9.76	9.94

No differences in total content are found between test formats, genders or native vs. non-native English speakers.

**Figure 3:**
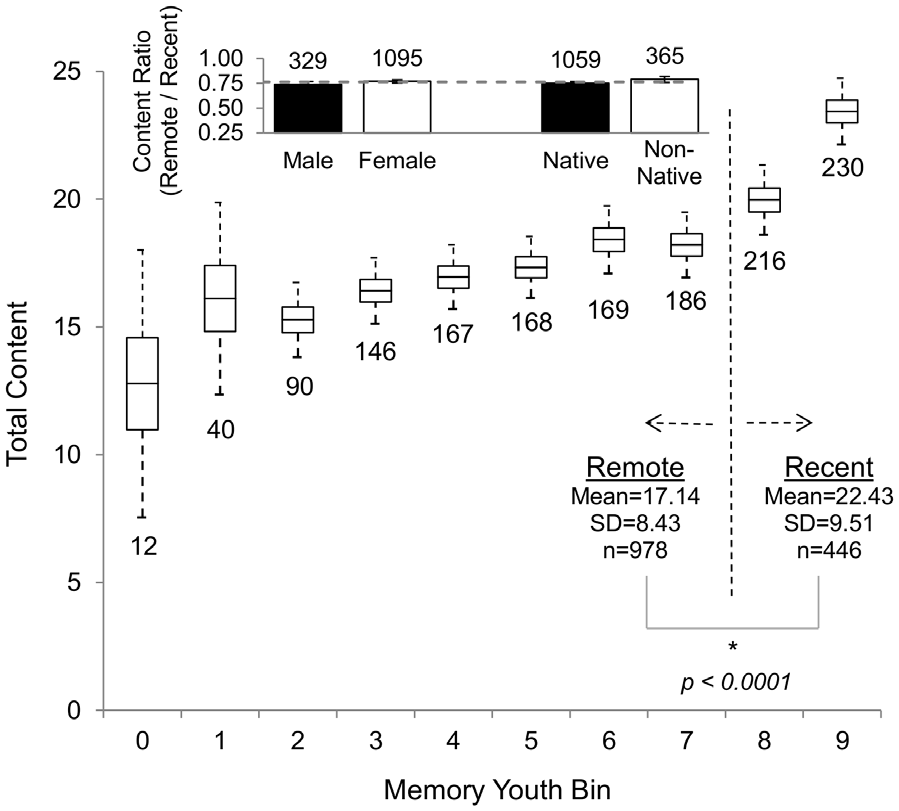
Total content across youth. Total content increases for memories retrieved from the first to the last tenth of life. Boxes represent the mean, 25^th^, and 75^th^ percentiles, while whiskers indicate 95% confidence intervals. Scored memories are divided into remote (bins 0–7) and recent (bin 8 and 9) time intervals, which account for 45.4%, and 54.6% of all dated memories, respectively. The total content of recent memories is 31% greater than that of remote memories (*p*<0.0001). The ratio between the two, i.e. the memory content lost to temporal degradation, is shown in the inset as the average (dotted line) and for two subject partitions as mean ± standard error.

The total content of AMs varied as a function of the age of the memory, with AMs recollecting recent episodes typically containing more elements than those retrieved from the more remote past (Fig. 3). However, the reduction of content with time appeared to be less compared to the reduction in the probability of memory recall. For example, a memory from the most recent past (bin 9) had only 40% more elements (∼23 vs. ∼17) than one from a middle period (e.g. bin 4). In contrast, the retrieval probability from the same examples (Fig. 2) was more than 400% greater (38.2% vs. 7.5%). Similarly, the ratio of total content from bins 8 and 1 (which for a 20 year old subject corresponds to episodes that occurred at ages 17 and 3, respectively) was 1.25, compared to more than a 10-fold factor in the retrieval ratio.

AMs were operationally divided into “recent” and “remote” halves, corresponding to the last two and first eight bins, respectively. This approximates a median-split [66], [67] with recent and remote AMs accounting for ∼55% and ∼45% of all dated recollections, respectively. Recent memories contained an average of 5 more elements than remote AMs, *t*(1422) = 10.54, *p*<0.0001, *r* = 0.27. This temporal effect was consistent across males, females, native, and non-native English speakers (Fig. 3 inset). To assure that this effect was not due to an arbitrary division of temporal periods, analyses were performed using various separations, e.g. between bins 8 and 9 or between bins 6 and 7, and provided equivalent results: *t*(1422) = 8.88, *p*<0.0001, *r* = 0.23; and *t*(1422) = 7.83, *p*<0.0001, *r* = 0.20, respectively.

### Some Features are More Memorable than Others in Remote and Recent Memories Alike

**Figure 4:**
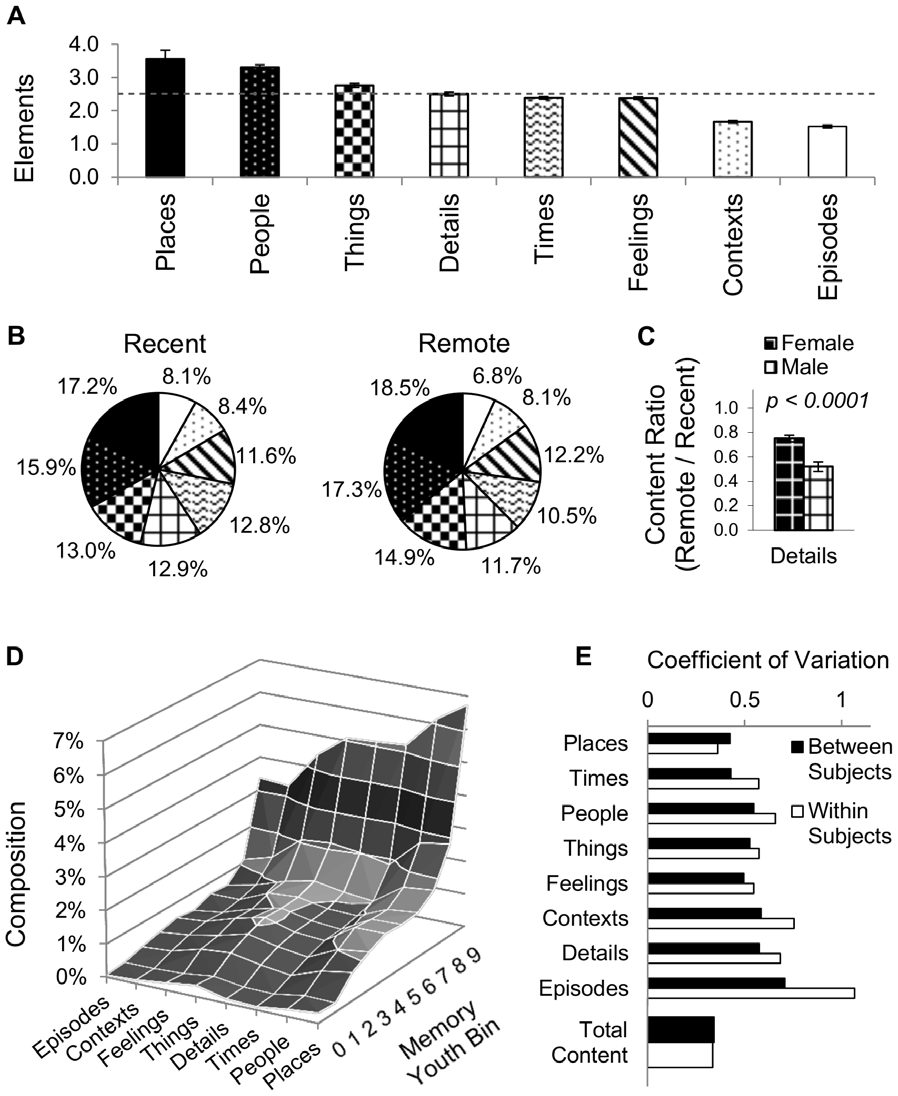
Feature composition of memory content. (A) The count of elements in each of the eight features is reported as mean ± standard error over all scored memories. More elements pertaining to Places and People are recalled than those pertaining to Contexts and Episodes. The dashed line represents the average number of elements (2.5). (B) The relative feature composition of recent and remote memories is similar (the slices are ordered by the recent rank). Although Feelings shifts from 4^th^ to 6^th^ rank from the remote to the recent distribution, the actual value change is very modest. (C) The content ratio (remote/recent) across all features was similar between genders with the only exception of Details, for which the decay was significantly greater in males (light hatching) than in females (dark hatching). (D) Recall composition of elements and youth in a typical AM. (E) For all features except one, the variability in the number of elements within subjects is greater than that between subjects. However, variability of total content is similar within and between subjects.

The overall content of AMs was analyzed in terms of the number of elements in each individual feature. This breakdown reveals two features that are particularly memorable, *Places* and *People*, each with more than 3 elements counted in the average memory. In contrast, *Contexts* and *Episodes* have fewer than 2 elements per memory each. The other four features have a number of elements per memory that remains close to the overall average of 2.5 (Fig. 4A). Interestingly, although the total content decays with time, the relative feature composition of AMs remains considerably stable from the most remote to the most recent memories (Fig. 4B); an equivalent pattern of feature composition emerges upon individual bin analysis. In particular, the relative ranking of the eight features remains largely unaltered, with *Places* being the most and *Episodes* the least represented features in both recent and remote memories. Together, *Places*, *People*, and *Things* amount to approximately half of the counted elements in recent and remote memories alike. In general, the ratio of the number of elements between remote and recent AMs for each and every feature remained close to that of overall content.

The overall feature composition of AMs was found to be remarkably similar between males and females. Although females remembered slightly more *Feelings* (2.5±1.7 vs. 2.1±1.5) and fewer *Details* (2.4±2.1 vs. 2.7±2.6) than males, these differences were not statistically significant, and the number of elements for all other features differed by less than 10% between genders. Similarly, the temporal decay, reflected by the remote/recent ratio was similar between female and male subjects across all features with the one noticeable exception of *Details* (Fig. 4C). Specifically, relative to the overall content decay of 0.76 (Fig. 3 inset) females tended to retain significantly more *Details* than males (ratios of 0.75±0.90 vs. 0.52±0.72, *t*(1422) = 4.25, *p*<0.0001, *r* = 0.07).

Combining the temporal and feature distributions, it is possible to compute the typical composition of recalled memories (Fig. 4D). Elements of *Places* from the most recent tenth of one's life are over 100 times more represented in AMs than elements of Episodes from the most remote tenth (6.7% vs. 0.06%). *Feelings* from bin 7 are approximately as likely to be recalled as *Things* from bin 4 (∼1%). Interestingly, for most features, content varied more among different memories of individual subjects, than across subjects. In contrast, the variability of total content was essentially identical within and between subjects (Fig. 4E). Altogether, these findings suggest that individual memories can vary substantially in their feature composition (e.g. one retrieved memory might have richer information on *Times* than *Contexts*, and another just the opposite), yet these effects tend to average out when considering all combined content and/or a large pool of memories.

A certain amount of correlation among the number of elements in the various features is expected, as richer memories are likely to have more elements in several features. However, some features may be more “independent” than others. In order to identify these more “fundamental” features, we computed the cross-correlation among features, as well as the correlation of each feature with all other content (Table 2). Interestingly, *People* and *Places*, the most memorable features, are also the least correlated with the rest of AM content, but *Episodes* (the least memorable) ties for second in this ranking, while *Feelings*, *Details*, and *Times* (the three features with average memorability) are last. These correlation values were essentially identical for males and females (data not shown).

**Table 2:** Feature Cross-Correlation.

		1	2	3	4	5	6	7	8
1.	People	-							
2.	Places	0.17	-						
3.	Episodes	0.22	0.16	-					
4.	Things	0.21	0.37	0.21	-				
5.	Contexts	0.18	0.24	0.31	0.26	-			
6.	Feelings	0.24	0.29	0.29	0.39	0.39	-		
7.	Details	0.26	0.26	0.38	0.30	0.39	0.36	-	
8.	Times	0.20	0.44	0.38	0.28	0.38	0.31	0.34	-
	**All Other Features** a	**0.33**	**0.43**	**0.43**	**0.46**	**0.48**	**0.52**	**0.52**	**0.52**

The numbers of elements in each feature are all positively correlated with each other across memories, as quantified by Pearson's correlation coefficients.

a The last row reports the correlation of each feature with the sum of the elements from all other features combined. Elements pertaining to *People*, *Places*, and *Episodes* are more independent, as indicated by lower correlation coefficients, while elements pertaining to *Times*, *Details*, and *Feelings* are more interdependent. The mean Pearson Coefficient across features with All Other Features is 0.46.

### Reminiscence of AMs Occupies a Substantial Fraction of Cognitive Time

**Figure 5:**
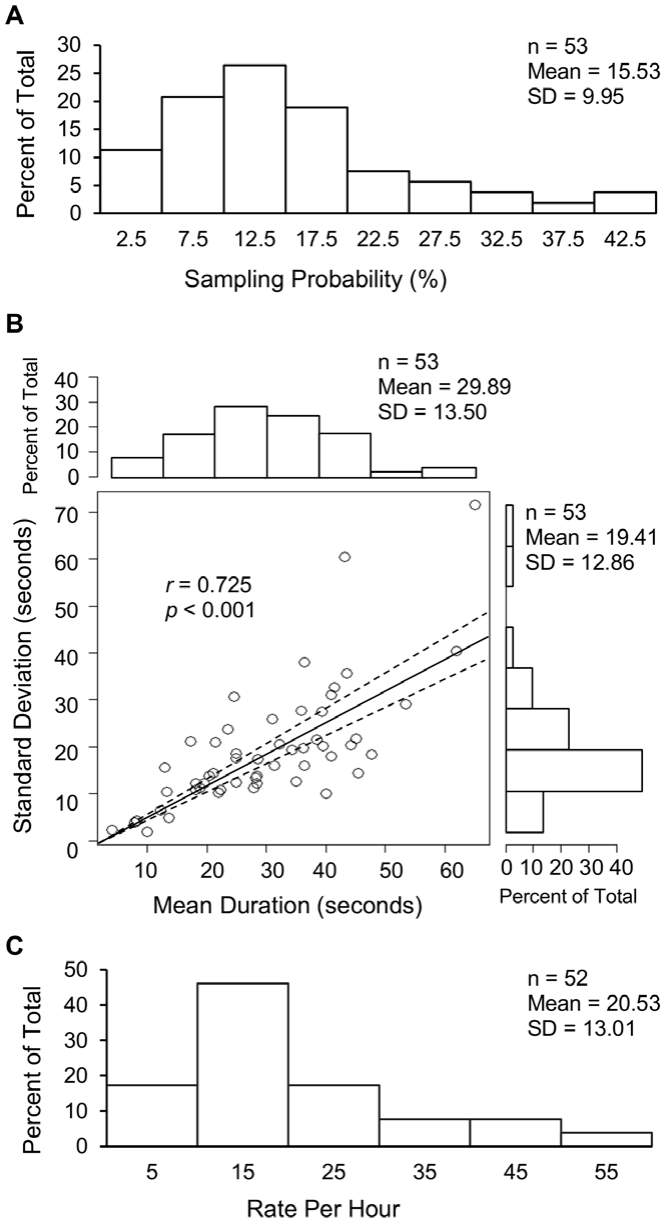
Frequency and duration of autobiographical memories. (A) The Spontaneous Probability of Autobiographical Memories (SPAM) was sampled on a participant-by-participant basis by dividing the number of memories reported by the total phone call prompts. (B) The mean and standard deviation of AM duration was computed for each subject over an average number of memories per subject of 48.5 (the standard deviation of this number was 29.3, range 3–133). Mean durations are directly and significantly correlated with the standard deviation. The slope of the best fitting line indicates a coefficient of variation of 0.65 with a 95% confidence interval of ±0.07 (dashed lines). (C) The number of AMs experienced per hour was calculated for every subject by multiplying his/her sampling probability by 3600 and dividing the result by the same individual's mean duration in seconds.

The SPAM protocol measured the rate at which AMs are recalled under normal conditions. On average, one in seven subjects reported reminiscing an AM at the time of a phone call. This corresponds to a mean fraction of time spent reminiscing of ∼15% (median: 14%, mode: 15%). However, this sampling probability varied considerably among individuals, ranging from less than 2% of the calls for some subjects to more than 40% for others (Fig. 5A).

The subjective estimation of the duration of AMs varied considerably both within and between subjects. In particular, the length of memory recall, averaged in each individual over the “positive” cases in which a phone call interrupted reminiscence, ranged from less than 5 to more than 60 seconds, with a grand mean around half a minute (median: 28 s, mode: 28 s). The standard deviation of this mean across individuals was 13.5 seconds. There was no correlation between the mean and the coefficient of variation of memory duration from subject to subject (Fig. 5B).

The probability of recall and memory duration data were used to compute the number of AMs retrieved in an hour. Such estimation yielded a right-tail skewed distribution, with an average of 20.5 AMs recalled per hour (median: 17, mode: 11). Except for one outlier, the range across individuals was 2 to 54 memories per hour. None of these metrics (i.e. probability, duration, and rate per hour) varied significantly between males and females or native and non-native English speakers (Table 3). Moreover, no recall differences were found within (i.e. early and late) or across (i.e. initial and subsequent, weekday and weekend) days of sampling (data not shown).

**Table 3:** Quantification of the spontaneous occurrence of autobiographical memories.

		Gender		English	
		Female	Male	Native	Non-Native
	n	29	24	41	12
Sampling Probability (%)	Mean	15.35	15.74	14.03	20.65
	SD	10.22	9.83	8.70	12.48
Memory Duration (s)	Mean	28.34	31.75	28.34	35.15
	SD	12.89	14.25	13.97	10.61
Rate Per Hour	Mean	21.10	19.88	20.12	21.92
	SD	14.19	11.76	13.47	11.77

The probability of occurrence, duration, and hourly rate are not significantly different between males and females or between native and non-native English speakers.

### Quantitative Estimates of AM Content Retrieval by Feature and Youth

**Figure 6:**
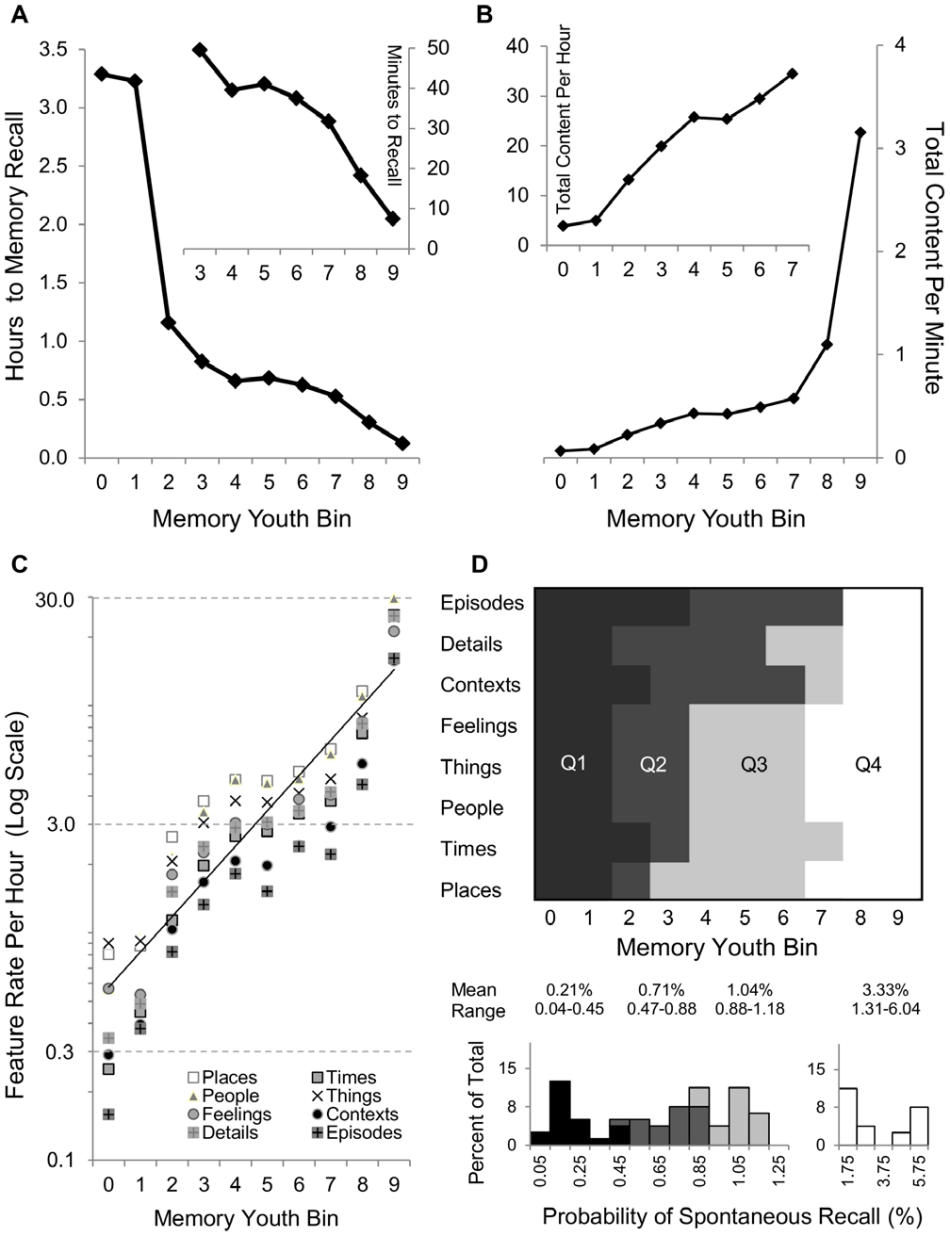
Retrieval probability of memories and features across youth. Integration of CRAM and SPAM data allows a number of quantitative estimates. (A) The average time elapsing between two memory recalls (e.g., more than 3 hours for the most remote AMs, but less than 10 minutes, see inset, for the most recent AMs). (B) The average recall rate of total content (e.g., more than 3 total elements per minute from the most recent tenth of life, but less than 3 per hour from the most remote tenth of life, see inset). (C) The number of elements for each feature recalled in one hour from each life period (the linear fit in the log scale, indicative of a power function, represents the average across features). (D) Momentary probability of recalling at least one element of a particular feature from a distinct bin, grouped in quartiles.

Data from CRAM and SPAM were combined to estimate the quantitative profile of subjective content in naturally-occurring AMs. Such analysis assumes that the temporal distributions and feature content assessed with CRAM on word-cued memories is sufficiently similar to that expected of AMs recalled in everyday life. While this vital assumption remains to be tested, such integration yields a useful baseline of quantitative hypotheses about probability of feature recollections from distinct life periods. In particular, the measured temporal distribution of cued memories, together with the spontaneous retrieval rate, allows the computation of the average period that elapses between recalls of AMs from a given bin (Fig. 6A). According to this analysis, a typical subject recalls a memory from the first fifth of one's life every ∼3 waking hours or ∼5 times a day. In contrast, only minutes separate consecutive retrievals of AMs from the most recent tenth of one's life.

In the same vein, total content retrieval can be computed as the number of elements recalled from various bins per unit of time. A total of 412 elements are recalled per hour (7 per minute, or one every 9 seconds on average); 62% of this amount is from the recent past, i.e. from when a 20 year old subject was 17 or older (Fig. 6B). This analysis can be further broken down by individual features (Fig. 6C). For example, while 3 contextual elements are recalled in an average hour from bin 7, the same rate of 3 features per hour holds for the number of recalled places from the more remote bin 2. Moreover, one may estimate the probability of recall, at any given time, of at least one element of a particular feature from a specific period of his/her life (Fig. 6D). For example, these results indicate that in any given moment, approximately one in 167 awake people (0.60%) are experiencing an AM containing an object (*Things*) from their third tenth of life, while ten times as many individuals (6%) are recalling a place from their most recent tenth of life. More generally, these data may be summarized by dividing all 80 combinations of 8 features and 10 bins in quartiles based on the relative probability that at least an element of the corresponding pair would be recalled at any one time. The first (i.e. least probable) 3 quartiles are uniformly distributed in decreasing order of the age of the memory, as indicated by the respective probability means and ranges (Fig. 6D, bottom histograms). In contrast, the fourth (most probable) quartile introduces a discontinuity (from the 0–1% to the 2–5% range) displaying a bimodal distribution, clearly reflecting the retention effect.

## Discussion

This work begins to answer two open yet integral questions in the scientific characterization of AMs, i.e. the types and numbers of elements that comprise their content, and their frequency and duration under natural conditions. Specifically, for typical everyday AMs, what features are recalled and how many? From when are the recalled events, and how often are they remembered? In order to tackle these questions, we introduced two novel tools, the CRAM (Cue-Recalled Autobiographical Memory) test and SPAM (Spontaneous Probability of Autobiographical Memories) protocol. Compared to commonly used methods, these procedures permit more time-effective data collection and comprehensive analysis of AM content and occurrence. The results reported here complement and expand existing knowledge on the temporal distribution, subjective content, and frequency of AMs. This is accomplished by quantifying the number of elements retrieved in specified features from naturalistically-cued AMs of numerous life periods in addition to the spontaneous retrieval probability and overall occurrence of AMs during everyday life.

The seminal cue-word research design of Crovitz & Schiffman [18] opened a path to quantify the effect of AM age on its relative frequency. Several studies adopted this method in the popular variations of Robinson [19] and Rubin [21], which fixed the word set to maximize reproducibility. Specified qualities of AMs were also measured with rating scales (e.g. MCQ [12]), and in relatively few cases those aspects of content were adopted to count the number of details recalled [37].

CRAM combines an adaptation of the cue-word technique with a variation on feature-counting assessments by asking subjects to date retrieved episodes and to count the identifiable elements of their own AMs in each of several features. In order to emulate everyday cuing instances, we chose to sample cue words stochastically based on their usage frequency in natural language. Moreover, to further mimic naturalistic conditions of memory retrieval, we prompted the subject with a list of 7 words instead of just one. Consistent with previous reports (e.g. [11], [20]), we observed a retention effect for the most recent AMs, a power decay for intermediate AMs, and childhood amnesia for the most remote AMs. This temporal distribution of retrieved episodes was also robust with respect to subject gender, native language, and details of the computer interface.

To the best of our knowledge, this is the first report of the absolute number of elements retrieved in AMs using cues based on their natural usage frequencies. Both the mean value (∼20) and the coefficient of variation (∼0.5) were equivalent between genders, native language, and computer interface. Moreover, total content values collected with CRAM are similar to those reported when cuing memories with typical life events and scoring event narratives [37]. Those same previous studies showed that experimenter probing of specified feature categories may aid retrieval of content [37]. Nevertheless, the amount of total content collected through CRAM closely resembles that obtained without such specific probes, suggesting the counted details are more akin to those recalled spontaneously. This apparent conflict may be reconciled by highlighting methodological differences. CRAM provides description and examples of what constitutes an element in general feature categories, which differs from overt requests of an extensive number of item (or element) categories used in those earlier approaches.

Adding to previous studies, the combination of a naturalistic cue-word technique with content counts enabled analysis of how the content of everyday occurring AMs varies by the age of the memory. Total content was found to decay temporally, but not as prominently as observed in the temporal distribution of AMs. For example, AMs from the most recent two tenths of one's life are nearly five times more likely to be retrieved than AMs from the most remote eight tenths, but their total content is only 30% greater. Further breakdown of AM content composition revealed certain features (*Places* and *People*) that are generally more memorable than all others in both remote and recent memories. *People* and *Places* were also determined to be the most “independent” features, i.e. those least correlated with other features. Moreover, these two features had a relatively high ratio of elements recalled in remote vs. recent AMs, indicating resilience to temporal degradation. These results complement previous observations that memory cues pertaining to the “what” of the event produce the greatest amount of recalled details [68]. If AMs are supported by an underlying skeleton of core features, these data suggest *Places* and *People* as likely candidates for such a core. Barsalou [33] reported that cues for people and locations elicit AMs more quickly than other cues (e.g. time), possibly due to the underlying organization of AM. Similarly, our findings may relate to the notion that the number of details recalled in distinct features corresponds to how an AM is organized for retrieval (e.g. by location, person).

The variability of total content from memory to memory within a participant was similar to that of the content average over memories from subject to subject. In contrast, the variability of the content of individual features was greater among memories of a single subject than among all subjects. This finding reflects the intuitive expectation that different memories could have different composition, emphasis, and themes, while maintaining a consistent amount of retrievable information. Thus, one feature (e.g. *People*) might be richly represented in some episodes of one's autobiographic life yet absent in others. This expectation is consistent with other empirical reports. For example, in assessing the effects of emotional arousal on AM composition, Berntsen [47] found a greater proportion of central details recalled from shocking compared to happy AMs, but an equivalent total number of details.

Similar to the temporal distribution, total content, feature composition, overall content decay, and feature cross-correlation were all similar for males and females (as well as for native vs. non-native English speakers and between computer interfaces). The only aspect that discriminated between genders was the difference in temporal decay of the number of other *Details* between recent and remote memories, which was more acute in males than in females.

As the CRAM techniques differ in certain aspects from previous studies, it is appropriate to discuss those design choices and their implications. Numerous definitions of AM have been employed in previous research. While temporal and spatial specificity are commonly used criteria, the rules used to segment recalled episodes vary. To facilitate counts of AM content, the definition of AM provided here constrained the recalled episode to a few seconds. As the temporal distributions and content measures replicate previous research with different segmenting rules (e.g. [37]), on average this constraint does not seem to bias the recollections elicited along those dimensions.

CRAM utilizes sets of word cues that replicate natural language frequency to elicit recollections more comparable to those occurring in real life. As word cues were sampled randomly from the British National Corpus, the use of an international subject pool dictated monitoring of differences in AM recall between subjects of varying native languages. Given the absence of differences between native English and non-native English speakers and the lack of complaints or questions about particular cue words by any participant throughout the study, we surmise that CRAM in its current form is suitable for international users. As non-linguistic cues (olfactory, auditory, kinesthetic, pictorial, etc.) may also evoke AMs, the precise degree to which these word-sets mimic natural cuing experiences remains an open question. Since AMs elicited in the lab by varying sensory experiences have equivalent temporal distributions and vividness ratings [30], stricter use of naturalistic cues might not affect measures of AM content across the life span. Nonetheless, further investigation into the proportion of AMs elicited by various sensory experiences and the resulting qualities and quantities of recollection is warranted.

The CRAM protocol does not reveal which word or collection of words elicited retrieval. This drawback impedes cross-sectional comparisons within and between participants as in experimentally-controlled fixed-cue recollections. This limitation reflects the unavoidable tradeoff between lab conditions and naturalistic approaches. In principle, cross-sectional comparisons focusing on naturally-occurring AMs are possible by statistical analysis of very large sample sets.

CRAM collects counts of details within feature categories instead of extracting details from participant narratives. While it is unknown how participants score content in CRAM (e.g. by enumeration or approximation), a smooth count distribution was found for each feature (data not shown), suggesting that the results are not significantly distorted by rounding bias [69]. Furthermore, during pilot experiments to determine the effectiveness of instructions, test subjects were asked post-test to provide a description of the details counted for each scored AM. In each case, this detail-by-detail event description corroborated the number of details provided. While such examples do not explicitly identify a scoring strategy, they are consistent with the idea that counts collected through CRAM are representative of the subjective details comprising AMs.

SPAM revealed that subjects spent a substantial fraction of their day reminiscing AMs. In particular, when unexpectedly asked whether they were experiencing an AM, on average participants had a 15% probability of “being caught in the act.” Combined with the assessed duration of ∼30 seconds for a typical recollection, this result leads to the estimate of a mean recall rate of ∼20 AMs per hour. Additional investigations will be necessary to determine what proportion of the considerable inter-subject variability of these values (range: 2–54 AMs/hour) reflects genuine cognitive diversity and how much is due to experimental error or systematic bias. In particular, each subject had the prerogative to select the temporal windows to receive calls. This was necessary to avoid interruption of sleep, privacy, or professional activities such as class attendance. If the times people are willing to entertain unexpected phone calls are also well suited for reminiscence, this protocol would tend to overestimate the occurrence of AMs. Moreover, although participant instruction was delivered systematically, differential interpretation could underlie the resulting disparity among subjects.

The integration of CRAM and SPAM data enabled interesting estimates of the number of elements retrieved in a fixed time for each feature from a specific life period. Although such detailed inferences demonstrate the potential of these novel research approaches, future studies will have to verify the underlying assumptions.

We stress that this research design focuses on the **subjective** aspect of AMs. In particular, the exact meanings of the eight features, as well as that of autobiographical memory, are taken to consist of the subject's interpretation of the corresponding definitions and accompanying instructions. Thus, by construction of the research protocol, these empirical measurements reflect what a subject considers to be a *feeling* or a *context*, given the definitions and examples communicated in the briefing sessions. In particular, our data do not discriminate between “true” or “false” memories, because the analysis targets mental representation of autobiographic episodes rather than their historical occurrence in the material world (e.g. [34]). Moreover, we are measuring the subjective recalled content, independent of the total content that could possibly be recalled from those past episodes. Similarly, reminiscence duration in SPAM consists of subjective time estimates [70]. At the same time, both the selection of features and the specific wording adopted in the explanations and interactions with subjects were chosen in the course of extensive pilot studies on the basis of spontaneous suggestions from participants and debriefing interviews. In this sense, the analyzed features should in fact correspond to observable aspects of subjective experience.

This research utilized a college-aged subject pool, a common practice in AM research [9], [12], [13], [18], [19], [21], [30], [51], [59], particularly when exploring novel measures. As such, these data help provide the basis for comprehensive characterization of AM and quantitative testing of AM models [54]. With this aim, these methods are currently being employed with a larger and more diverse pool of subjects of many ages. The high correspondence in data collected between computer interfaces outlined here suggests that CRAM produces reproducible results. Ongoing collection of additional data will help determine the extent of variation due to different testing conditions (e.g. administered in person or online) and the applicability to participants of increasing age. The CRAM dating procedure assigns AMs to bins which vary according to the subject's age [61], [62]. While this normalizes the difficulty associated with dating AMs of increasing age [19], it creates discrepancies in the temporal ranges of two equivalent bins from younger and older subjects. To circumvent these discrepancies, when age is a central variable, analysis will require AM comparison both in terms of relative time periods (i.e. defined by participant-specific bins) [37], [61], [62] and absolute time periods (i.e. defined by the age of the participant and event) [37], [65]. Nevertheless, the precision associated with the absolute date of an AM will decrease with increasing participant age. Moreover, certain comparisons remain restricted, e.g. AM content from the most recent year of life from younger and older participants [37].

Given the benefits of CRAM and SPAM to quantify time-effectively the content and frequency of naturalistically sampled AM, these methods may be of value to cognitive and clinical psychology. Normative characterization of a representatively large population should be useful in assessing the effects of particular conditions (fatigue, stress, psychotropic substances, etc.) or genetic variants on AM recall, as well as monitoring the progression of memory disorders (e.g., Alzheimer's disease, Korsakoff's syndrome, and anterograde/retrograde amnesia) in individual patients. Attempting cross-sectional analyses in cognitively impaired populations, however, demands consideration of key factors, including task difficulty, interpretation of instruction, and comfort level with technology [71], [72].

Further application of CRAM and SPAM could prove useful in clarifying several open questions about human recollection. It has been reported that ratings of vividness and reliving are not related with a higher retrieval probability observed in the reminiscence bump [65]. As expected from the age range of the subject pool, our temporal distributions do not show a reminiscence bump. However, applying CRAM to older populations may help elucidate possible relationships between feature counts and retrieval probabilities. In addition, AM has been theorized to have specific functions (e.g. directive, self, and social [8]), which were corroborated by empirical findings [9]. SPAM, by its current design, does not establish why sampled AMs are recalled. However, the protocol may be adapted to isolate the frequencies of functionally-distinct AMs, and explore how their everyday usage frequencies change with age. SPAM also holds promise to clarify the relationship between voluntary and involuntary recollection. As past voluntary recollection may prime involuntary recollection [73], a variation of SPAM could be devised to obtain the frequency correlation between these two forms of retrieval on a participant-by-participant basis. A similar variation could directly compare frequency and duration of recollecting past events and future intentions (retrospective and prospective memory, respectively).

The data reported here provide comprehensive measures of the content and frequency of naturally-occurring AMs over the life span. Moreover, with the tools introduced in this work, collection and storage of age-specific population statistics in a large-scale informatics database (http://cramtest.info) is underway using internet sampling. Altogether, these advances constitute a further step towards the inclusion of subjective mental content in the realm of quantitative, reproducible science [74], [75], enabling deeper and broader queries of human memory content.

## Supporting Information

Supporting Information S1 **The supporting information document provides an in-depth description of the methods used in the CRAM test and SPAM protocol.** This includes the precise text provided to CRAM participants to instruct memory cuing (A) and feature scoring (B–C), the processing details of words sampled from the British National Corpus (D) [76], [77], examples of data screening as applied to CRAM (E), and the precise text provided to SPAM participants to permit identification of everyday AMs (F).
(DOC)
