# Supplementary material for: Quantitative Measurements of Autobiographical Memory Content
Source: PLoS One. 2012 Sep 21;7(9):e44809. doi: 10.1371/journal.pone.0044809 (PMC3448603; doi:10.1371/journal.pone.0044809)
Supplement: Supporting Information S1 — The supporting information document provides an in-depth description of the methods used in the CRAM test and SPAM protocol. This includes the precise text provided to CRAM participants to instruct memory cuing (A) and feature scoring (B–C), the processing details of words sampled from the British National Corpus (D) [76], [77], examples of data screening as applied to CRAM (E), and the precise text provided to SPAM participants to permit identification of everyday AMs (F). (DOC) [file pone.0044809.s001.doc]

## Supporting Information

*A. Instructions for CRAM part 2 (word-cuing of AMs)*

In this part you will be prompted with a set of words. Your task is to recall the first autobiographical memory that the words bring to your mind. This means just the first memory that you think of, not the earliest memory. The episode may have occurred any time from birth to now. Your memory does not need to be associated in any way to the given set of words. It is also ok if the memory comes to your mind before you have finished reading all of the words in the set. This memory should be of a brief, self-consistent episode of your life. An episode can be as short as a single snapshot and up to a few seconds long.

After you experience a memory, write down either a word or a simple phrase in the text area provided below that will allow you to recall the specific memory later in the test, and press “Label Memory” to move on with the test. The notes you write are for your own use only; at the end of this test they will be deleted and not recorded anywhere. You should feel free to write any personal information. The purpose of these notes is just for you to recall this same memory later in the test. They should be as brief and informal as you like, so long as they positively identify the specific memory in your mind.

If the memory you think of refers to a typical and repeated episode that happened regularly or multiple times in your life, you can use it only if you can fixate on a specific individual event. If you can only recall the generic (repeated) event, look for another memory.

If after a few seconds you cannot retrieve any autobiographical memory after reading the set of words, leave the text space blank and click the “No memory comes to mind” button to produce a different set of words.

*B. Instructions for CRAM part 4 (Feature descriptions and examples)*

*People:* How many uniquely identifiable persons (excluding yourself) do you remember in the episode? Example: You were at some party. Your best friend John was there, and so was his second wife, whose name you don’t recall. The host, Marc, was there, and some of his relatives, but you cannot remember which. *Count 3 elements* (John, his wife, and Marc).

*Feelings:* How many distinct subjective feelings (tastes, odors, temperature, emotions, etc.) do you recall in the episode? Example: It was the last day of school. You had a stomachache, the room smelled like fish and it was too warm. Still, you felt very happy. *Count 4 elements.*

*Episodes:* How many other episodes that immediately precede or follow this one can you recall? Example: John remembers the first homerun he hit. He remembers the instance the ball hit his bat and a thunderous crack rang out. Recalling the episodes that led up to and following this moment, John recalls taking a warm-up swing before entering the batter’s box. He also recalls focusing on the pitch right before the ball was thrown. After he hit the ball he recalls running around the bases after which his memory fades but he recalls later that his team went out for a celebratory pizza. Though going out for pizza is a memory, it is not counted because it is not sequential - there is a gap in time. *Count 3 elements.*

*Places:* How many spatial features do you remember of this episode: town, house or road, room or vehicle, your exact position, etc. Example: You recall chatting with a friend in her apartment in New York, but not whether in the living room or bedroom, nor whether sitting or standing. *Count 2 elements* (for the apartment and the town, even if knowing the apartment “automatically” specifies the town).

*Things:* How many uniquely identifiable objects do you remember (must have at least one detail such as texture, material, size, color, or else be out of context)? Example: If you were inside a bedroom, the window doesn’t count as an object (since almost all bedrooms have one), unless you remember that it was open, or that it had pink curtains… Same with a bed, a closet, etc. If, on the other hand, you remember there were skates on the floor, an apple on the table, or something not usually found in the standard bedroom, then *you should count those objects*.

*Times*: How many temporal features do you remember of this episode: the exact year, month or season, day of the week, time of the day, etc. Example: You remember getting a speeding ticket while driving to church. You can’t remember the exact year, nor time of the day, but you recall it was summer and Sunday. *Count 2 elements.*

*Contexts:* How many other explicit contextual details (weather, situations, events, etc.) do you remember? Example: You remember that on that same day, the Lakers’ won the league, your grandma was at the hospital, and it was freezing cold outside... *Count 3 elements*.

*Details:* How many other particular details do you recall (words uttered or heard, facial expressions, actions, clothing...)? Example: It was your first date. When you arrived, she said: “late at your first date!?”, and you smiled. She had already ordered a drink. *Count 3 elements.*

*C. Optional (hyperlinked) guidance on what constitutes “an element” for CRAM part 4 (feature count of AMs)*

A detail could be practically defined as the minimum element of information you would include in a very extensive and exhaustive account of this episode in a hypothetical personal diary. As with a personal diary, you would not describe over and over objects or people you are very familiar with.

Suppose that the episode consisted of an argument you had with an old friend in your kitchen. Even if you can probably visualize in your recollection many details of the kitchen, such as the position of the refrigerator, the color of the walls, and whether you had a gas or electric stove, these are not really part of the specific episode. You would not describe them in your diary, because they would be implied by the fact that the episode occurred in your kitchen. Thus, you should not count these details in the test. Similarly, you should not count the fact that your friend had blond hair and blue eyes. However, if the argument degenerated and the friend broke a dish on your head, you should probably count that dish as an object even if you had seen it many times before in the kitchen.

If, on the other hand, you are describing a hotel room you spent one night at, then every uniquely identifiable detail you can remember should count. You can’t, however, consider “the room had a door, a bed, and a lamp” as three valid details, unless you remember something specific about them.

If in another recalled episode you changed a flat tire, and remember that you had to unscrew as many as 16 bolts, should you count 16 details? Not unless you remember something specific for each and every bolt. In your diary you would probably write that there were 16 bolts, and this single element of information should be counted as one detail. Similarly, if you recall a dinner with 12 people, but only specifically remember 3 of them, you should count three details under “people” and one under “contexts” (corresponding to the fact that there were 12 people). If you remember that one person at dinner was a lawyer, but you don’t remember his face nor any other detail about him, should he count as a person? You can count him in, or alternatively you could count the fact that one person in the group was a lawyer as an “other” detail (it would in any case count as one detail overall).

In general, there is no objectively “right” or “wrong” way to exactly count details in a remembered event. What matters most is what you consider a detail in your memory, and as such you are the ultimate decision maker. No need to agonize over the specific category of the element. The distinctions between various categories are often ‘soft’, and you can decide just based on your intuitive preference.

*D. Processing of the British National Corpus to sample cue-words*

The initial corpus (http://www.natcorp.ox.ac.uk) [60] consists of adjectives, cardinal and ordinal numbers, proper and common nouns in all their forms, and non-auxiliary verbs from the “demographic” (i.e. conversational) file. Articles, conjunctions, and prepositions, such as “and”, “the”, and “of” were excluded from this master list. A set of 187 obscene or otherwise questionable terms was identified with a freeware word filter tool ([http://www.discusware.com](http://www.discusware.com/)) [76]. Potentially offensive terms were removed from the word pool including (but not limited to) the original and all derivatives of the seven terms that the U.S. Supreme Court ruled cannot be used on television (FCC v. Pacifica Foundation, 438 U.S. 726, Decided July 3, 1978) [77]. Finally, words with a usage count (as reported in the British National Corpus) of 4 or less out of 108 were also excluded to avoid arcane terms. The remaining list consisted of 13,241 distinct terms with an average usage frequency of 90 times for every 108 words. Cue-words were sampled with a weight proportional to their usage frequency, with the additional constraints that repeated words, the plural and singular forms of the same regular nouns, and the first- and third-person forms of the same regular verbs could not be re-sampled within the same test. An example of a randomly sampled set of 7 words was: “noise, abrupt, cashier, belt, juice, flee, shells.” The number of times that these words appear in the British National Corpus is 215, 11, 7, 109, 136, 6, and 10 times (out of 10^8^), respectively.

*E. Representative examples of data screening*

One suspicious pattern was detected in the AM temporal distributions from one participant, who dated all memories into the first (most remote) time bin. These data were excluded from analysis. Unusual entries were noted in the analysis of memory content, and screened based on the variability within and between subjects. In particular, outliers were identified at the level of individual participants and of individual memories. As a real data example, one memory was found to have a feature count of 100 elements in the People category, far beyond what could be explained by the variance observed in the remaining memories scored by that participant, who recalled a median of 2 people per AM. The entry was marked as a possible mouse slip and the individual memory was excluded from analysis. In a second case, a participant systematically displayed repeated counts among the eight features in the majority of AMs (e.g. one memory had 3 elements in each and every feature, while another had zero elements throughout, etc.). All memories from this subject were excluded from analysis.

*F. Examples of Autobiographical Memories included in the SPAM instruction packet*

An autobiographical memory (AM) refers to an episode of your personal past; a memory of something that you have personally experienced in your lifetime. This memory could be of an event that occurred from the very moment you were born to the last second you just lived. The event in the memory is typically specific to a place and a time.

*NO (Not An) AM Example:* You remember a 3 hour long trip to your grandmother’s house and thinking how much longer it felt with an annoying little sister to share the time with.

*Why Not?* This memory would not to be considered an AM because the memory is of an event that was 3 hours long.

*Yes (An) AM Example:* You are remembering that once on a 3 hour ride to your grandmother’s house, you saw a cow for the first time. You can see the cow again through your mind’s eyes and you remember what you felt when you saw the cow (curious or afraid for example) and the smell of manure, mmmh.

*Why?* This is an AM because the event that you are remembering is specific to a moment in your life. That is, this event only lasted a few minutes, and did not stretch out over 3 hours, days, weeks, etc. Also, re-experiencing the feeling and smell of that event is a sign of mental time travel, a hallmark of AMs.

*Yes (An) AM Example:* You are remembering the moment your dog ran through the door of your home after being lost for two days. You are re-living the feeling of happiness and relief that Murdock (your dog) is safe and licking your face like a giant lollipop. You can actually feel his wet tongue on your face, that’s how much you’re into this memory.

*Why?* This is an AM because you are obviously re-living some portions of this event (i.e. emotional and physical feelings). Also, this memory is of an event that happened personally to you.

*NO (Not An) AM Example:* You remember a story about a person who for two days hopelessly looked for her dog, when a neighbor finally brought it over to her. After about a week of giving her dog extra love and extra long walks to make up for lost time, she realized that this was not her dog.

*Why Not?* This is not an AM because it is not an event that happened to you, this is a memory of an event from someone else’s life.

*NO (Not An) AM Example:* You remember that for the past week, every time you turn your head fast you get a shooting pain down your spine. You can still feel what this pain was like.

*Why Not?* This is not an AM because the event is repetitive; you have experienced this event several times in your life.

*Yes (An) AM Example:* You remember that for the past week, every time you turn your head fast you get a shooting pain down your spine. Specifically, you remember the first time that it happened, when someone called out your name and you jerked your head around to see who it was.

*Why?* Though this is a memory of an event that has happened several times over your life, you are remembering one specific instance that happened.

*Your Turn*

*YES or NO?* You remember that earlier today, when you were heading out for lunch you saw a deer that seemed so calm that you could have pet it. You feel nervous just remembering this situation.

*YES or NO?* You remember when you were a child, every 4th of July you went to your grandparents’ house and they always had your favorite dish prepared for you. You remember the smell and the taste of it, maybe your mouth is watering.

*YES or NO?* You remember last year your car broke down and you swore to your car that you were going to sell it. You remember how frustrated you felt. You remember that you popped the hood of your car to find out that it was only a loose battery cable. You re-experience the relief you had when you found this out. You remember getting into your car and apologizing for yelling at it.

*YES or NO?* You remember that you went to Jefferson High school and that your locker combination was 23-44-02.

*YES or NO?* You remember a story that your parents always tell about you whenever you bring a date home. The story is about when you were 10 years old and got so scared that you wetted your pants, how embarrassing.
